# New record of the cockroach genus *Pseudophoraspis* (Blaberidae, Epilamprinae) from China with descriptions of three new species

**DOI:** 10.3897/zookeys.273.4122

**Published:** 2013-02-27

**Authors:** Zongqing Wang, Keliang Wu, Yanli Che

**Affiliations:** 1 Institute of Entomology, College of Plant Protection, Southwest University, Beibei, Chongqing 400716, China

**Keywords:** Dictyoptera, Blattodea, taxonomy, species group

## Abstract

The genus *Pseudophoraspis* Kirby, 1903 with three new species, *Pseudophoraspis clavellata* **sp. n.**, *Pseudophoraspis recurvata* **sp. n.** and *Pseudophoraspis incurvata* **sp. n.**, are reported from China for the first time. This extends the range of this genus northward from Vietnam. Species studied in the present paper are illustrated and described, and a key to these species based on males is provided.

## Introduction

The genus *Pseudophoraspis* was erected by Kirby in 1903. In 1904, he transferred *Epilampra congrua* to *Pseudophoraspis*. Shelford (1910) synonymized *Pseudophoraspis congrua* (Walker, 1868) with *Pseudophoraspis nebulosa* (Burmeister, 1838), and transferred *Epilampra miranda* Shelford, 1906 and *Homalopteryx vasta* (Walker, 1868) to *Pseudophoraspis*. Meanwhile Shelford (1910) also described the species *Pseudophoraspis* *fruhstorferi*. Bruner (1915) disagreed with Shelford’s views on the taxonomic status of *vasta* and returned it to *Homalopteryx*. Princis (1958) pointed out that the species *vasta* should belong in the genus *Pseudophoraspis*. However, Princis (1967) assigned *vasta* to *Rhabdoblatta*. Bruijning (1948) described *Pseudophoraspis proximata* and transferred *nebulosa* to *Stictomorphna*; later Princis (1967) transferred them to *Rhabdoblatta* and *Stictolampra*, respectively. Hanitsch (1923, 1925, and 1933) described four species assigned to this genus: *Pseudophoraspis emarginata*, *Pseudophoraspis testudinaria*, *Pseudophoraspis lacrimans* and *Pseudophoraspis uniformis*. Of these, one species, *Pseudophoraspis emarginata* wastransferred to *Stictolampra* by Princis (1967), and he only listed 6 species in this genus in *Orthopterorum Catalogus*. Anisyutkin (1999, 2005) added 9 new species to this genus from Southeast Asia. So far, the genus *Pseudophoraspis* is comprised of 15 species worldwide.

All of the known species were reported from Southeast Asia and South Asia, and the previously known boundary of this genus would be Vietnam. However, we found three new species from China, located in Hainan, Yunnan and Guangxi Provinces respectively. This discovery extends the range of the genus *Pseudophoraspis* northward. We redescribe the three known species, describe and illustrate the three new species, and provide a key for all species from China based on males.

## Materials and methods

Terminology used in this paper mainly follows McKittrick (1964) and Anisyutkin (1999). The genital segments of the examined specimens were macerated in 10% NaOH and observed in glycerin jelly using a Motic K400 stereomicroscope. All drawings were made with the aid of a Motic K400 stereomicroscope. All images of specimens were photographed using a Canon 50D plus a Canon EF 100mm f/2.8L IS USM Macro lens combined with Helicon Focus software. All specimens studied are deposited in the Collection of College of Plant Protection, Southwest University (SWU) and the Entomological Museum of Northwest Agriculture and Forestry University (NWAFU), as indicated.

## Taxonomy

### Genus Pseudophoraspis

Kirby, 1903 new record to China

http://species-id.net/wiki/Pseudophoraspis

Pseudophoraspis Kirby, 1903: 275; Kirby 1904: 119; Shelford 1910: 12; Hanitsch 1915: 72; Princis 1958: 65; Princis 1967: 660; Anisyutkin 1999: 444; Anisyutkin 2005: 40.

#### Type species.

*Epilampra nebulosa* Burmeister, 1838

#### Description.

Coloration brownish yellow, glossy and finely granulose (Figs 1–14). Pronotum smooth, broad and rhomboidal, completely covering vertex, anterior margin curved and posterior margin obtusely produced (Figs 1, 3, 5, 7, 9, 11, 13). Tegmina and wings in both sexes fully developed, entirely covering abdomen, apices rounded or posterior margin emarginate in the middle (Figs 1-14). Pulvillus and arolium present; tarsal claws symmetrical and unspecialized. Supra-anal plate and hypandrium nearly symmetrical, posterior margin emarginate near the middle except in *Pseudophoraspis fruhstorferi* Shelford (Figs 15, 17, 23, 25, 30, 32, 39, 41, 49, 51, 59, 61). Sclerite *L2d* of male genitalia with well-developed apical outgrowth (Figs 34, 35, 43, 44, 53, 54, 63) except in *Pseudophoraspis fruhstorferi* (Fig. 19) and *Pseudophoraspis tramlapensis* (Fig. 27).

Females usually shorter and wider than males, more convex, with smaller and widely separated eyes; pronotum larger. Tegmina and wings more or less shorter than in males (Figs 3–4).

According to original descriptions and examined specimens kept in Collection of College of Plant Protection, Southwest University (SWU) and the Entomological Museum of Northwest Agriculture and Forestry University (NWAFU), we subdivide this genus into two species groups: the *fruhstorferi* group and the *gorochovi* group. There are two types of pronotum in this genus: the pronotum of the *fruhstorferi* group is smooth and without any punctures, while in the *gorochovi* group, the pronotum is scattered with punctures and has two crescent depressions on disc.

**Figures 1–14.**
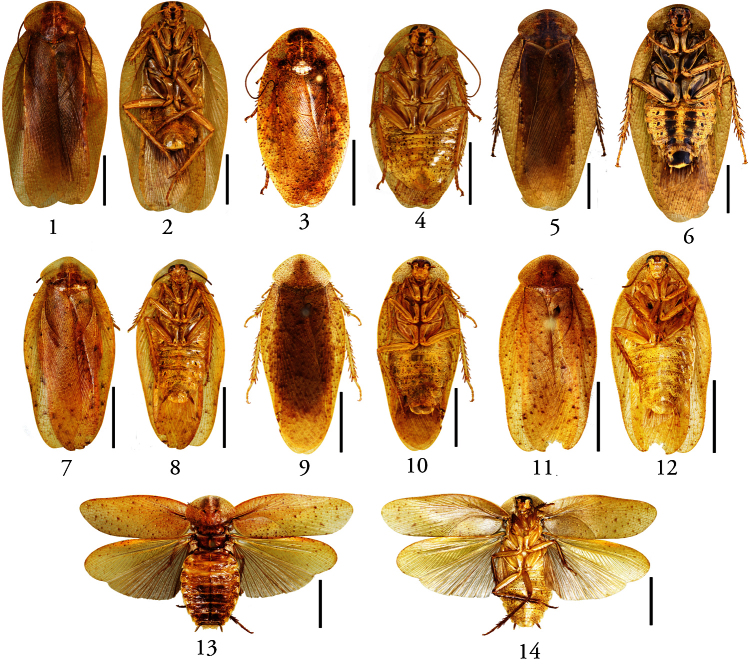
**1–4** *Pseudophoraspis fruhstorferi* Shelford, male: **1** dorsal view **2** ventral view; female: **3** dorsal view **4** ventral view; **5–6** *Pseudophoraspis tramlapensis* Anisyutkin, male: **5** dorsal view **6** ventral view; **7–8** *Pseudophoraspis kabakovi* Anisyutkin, male: **7** dorsal view **8** ventral view; **9–10** *Pseudophoraspis clavellata* sp. n., male: **9** holotype, dorsal view **10** holotype, ventral view; **11–12** *Pseudophoraspis recurvata* sp. n., male: **11** holotype, dorsal view **12** holotype, ventral view; **13–14** *Pseudophoraspis incurvata* sp. n., male: **13** holotype, dorsal view **14** holotype, ventral view. Scale bars=1cm.

#### Remarks.

The *gorochovi* group is similar to *Stictolampra* Hanitsch for the scattered punctures of the pronotum, but differs from the latter by the following characters: 1) pronotum rhomboidal, anterior margin curved and completely covering vertex, lateral sides approximately acute and angled, posterior margin obtusely produced and with two crescent depressions on disc; while in *Stictolampra*, pronotum peach-shaped, anterior margin approximately straight and vertex exposed completely, lateral sides more or less rounded, posterior margin strongly produced and without any depressions on disc; 2) sclerite *L2d* with well-developed apical outgrowth, while *Stictolampra* lacks any apical outgrowth.

#### Distribution.

China (Yunnan, Guizhou, Guangxi, Hainan); Indonesia (Sumatra, Java Island, Sulawesi); Malaysia (Malacca State, Borneo Island); Cambodia; Thailand; Vietnam.

#### Key to the species of *Pseudophoraspis* (males) from China

**Table:** 

1	Pronotum smooth without any punctures and depressions	2
–	Pronotum coarse scattered with fine punctures, and with two crescentic depressions on disc	4
2	Face with 1 large and 2 small brown spots. Sclerite *L2d* without apical outgrowth	3
–	Face without any spots. Sclerite *L2d* with apical outgrowth	*Pseudophoraspis kabakovi*
3	Apodema of complex *L1* long with folded terminal, sclerite *L2d* small, basal margin of sclerite *L2vm* transversal	*Pseudophoraspis fruhstorferi*
–	Apodema of complex *L1* short with transverse terminal, sclerite *L2d* large, basal part of sclerite *L2vm* furcated	*Pseudophoraspis tramlapensis*
4	Tegmina variegated with irregular brown spots. Apical outgrowth of sclerite *L2d* nearly straight	*Pseudophoraspis clavellata* sp. n.
–	Tegmina yellowish brown scattered with regular round brown spots. Apical outgrowth of sclerite *L2d* curved	5
5	Apical outgrowth of sclerite *L2d* bending outward	*Pseudophoraspis recurvata* sp. n.
–	Apical outgrowth of sclerite *L2d* bending inward	*Pseudophoraspis incurvata* sp. n.

##### *Pseudophoraspis fruhstorferi* group

Species included here:*Pseudophoraspis fruhstorferi* Shelford, 1910, *Pseudophoraspis tramlapensis* Anisyutkin, 1999 and *Pseudophoraspis kabakovi* Anisyutkin, 1999

### Pseudophoraspis fruhstorferi

Shelford, 1910 new record to China

http://species-id.net/wiki/Pseudophoraspis_fruhstorferi

Figs 1–4 15–22

Pseudophoraspis fruhstorferi Shelford, 1910: 12; Hanitsch 1927: 37; Bruijning 1948: 132; Anisyutkin 1999: 451.

#### Description.

**Male.** Body yellowish-brown. Head yellowish-brown, facial part of head with large brown spot from ocellus to clypeus, basal margin of ocellus with a round brown spot, eyes dark brown, ocelli yellowish (Figs 2, 4). Pronotum yellowish-brown, covered with scattered brown spots, disc part brown (Figs 1, 3). Tegmen yellow, one third of radius vein from base yellowish white (Figs 1, 3). Dorsal part of abdomen brown; ventral part of abdomen yellow, with dense small brown spots (Figs 2, 4).

Vertex completely covered by pronotum (Figs 1–2). Distance between eyes about 0.36 times of width of head. Ocellus same as scrobe and ocellus width equal to interocular width (Fig. 2). Pronotum rhomboidal, smooth and impunctate, much broader than long, anterior margin curved and posteriorly obtusely produced (Fig. 1). Tegmina and wings exceeding the abdomen and apex rounded (Figs 1–2). Fore femur with 4-7 spines along anterior margin and 2 apical spines. First segment of hind tarsus with spines along most part of its length and plantula occupying the terminal.

**Figures 15–38.**
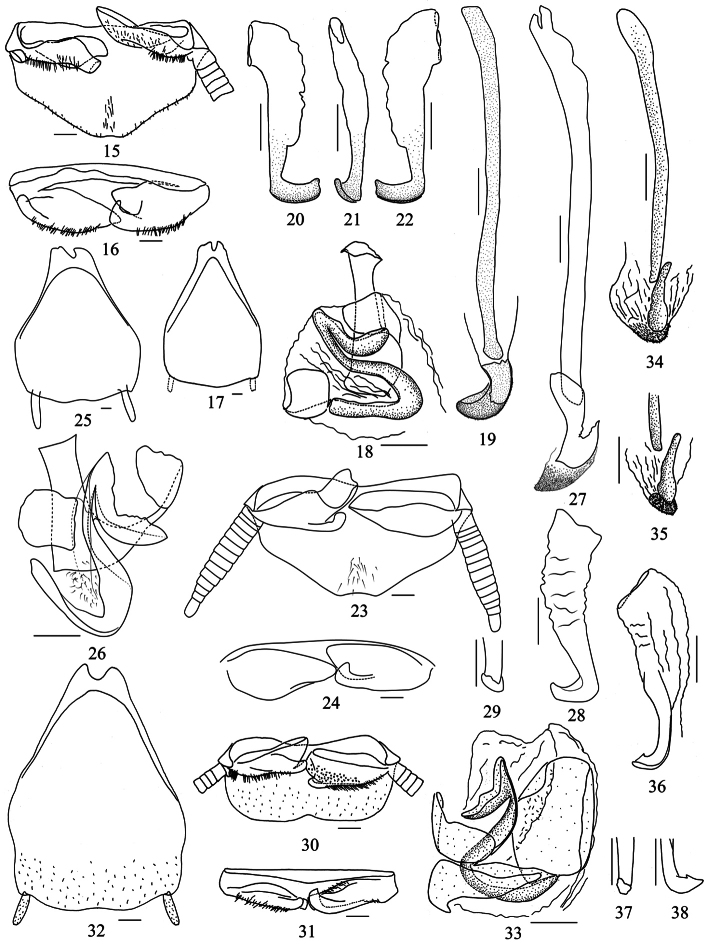
**15–22** *Pseudophoraspis fruhstorferi* Shelford **23–29** *Pseudophoraspis tramlapensis* Anisyutkin **30–38** *Pseudophoraspis kabakovi* Anisyutkin. **15, 23, 30** supra-anal plate, ventral view **16, 24, 31** paraproct, caudal view **17, 25, 32** hypandrium, dorsal view **18, 26, 33** complex *L1* **19, 27, 34, 35** complex *L2*, dorsal view **20–22, 28–29, 36–38** sclerite *R2*. Scale bars=0.5mm.

**Male genitalia.** Supra-anal plate symmetrical, trapezoidal, posterior margin with a shallow concavity at middle, lateral sides nearly straight (Fig. 15). Paraprocts asymmetrical, left one broad, simple plate; right one with a stubby finger-like protrusion whose apex bending backwards (Fig. 16). Hypandrium with posterior margin slightly produced, without any concavity (Fig. 17). Complex *L1* with apodema slightly long (Fig. 18). Sclerite *L2d* short, asymmetrical and densely covered with chaeta, without apical outgrowth, *L2vm* distinct and strongly sclerotized (Fig. 19). Sclerite *R2* with terminal rectangular and apex slightly elongate as a tooth (Figs 20–22).

**Female.** Usually shorter and wider than male (Figs 3–4). Eyes smaller and widely separated, distance between eyes about 0.39 times width of head (Fig. 4). Pronotum, tegmina and abdomen brownish-yellow, covered with scattered brown spots (Fig. 3). Facial part of head with large brown spot same as the male (Fig. 4). Dorsal part of body more convex than in male. Tegmina and wings shorter than the male, with posterior margin slightly emarginate (Figs 3–4).

**Male measurements.** Body length: 35.5–41.5 mm (including tegmen); Head length × width: 4.5–5.0 mm × 4.0–4.5 mm; Pronotum length × width: 8.0–9.0 mm ×11.5–12.5 mm; Tegmina length × width: 30.5–35.0 mm × 12.0–12.5 mm.

**Female measurements.** Body length:29.0–30.0 mm (including tegmen); Head length × width: 5.0–5.5 mm × 4.0–4.5 mm; Pronotum length × width: 8.0–8.5 mm × 11.5–12.0 mm; Tegmina length × width: 25.0–25.5 mm × 10.5–11.0 mm.

#### Material examined.

one male, China: Guangxi Prov., Mt. Daqingshan, 15 May 1963, coll. Yang Jikun (SWU); onemale, China: Hainan Prov., Mt. Jianfengling, 29 June 1981, coll. Lin Zai (SWU); two females, China: Hainan Prov., Mt. Wuzhishan (N18.51°, E109.40°) 740 m, 28-30 June 2008, coll. Zhang Weiwei (SWU).

#### Distribution.

China (Guangxi, Hainan); Vietnam.

#### Remarks.

Sclerite *L2d* of this species and *Pseudophoraspis tramlapensis* are strongly sclerotized and without apical outgrowth, but *L2vm* of the former with basal margin transversal.

### Pseudophoraspis tramlapensis

Anisyutkin, 1999 new record to China

http://species-id.net/wiki/Pseudophoraspis_tramlapensis

Figs 5–6 23–29

Pseudophoraspis tramlapensis Anisyutkin, 1999: 453.

#### Description.

Body yellowish-brown. Head yellowish-brown, the dark spots on facial part same as *Pseudophoraspis fruhstorferi* (Figs 5-6). Pronotum and tegmina densely scattered with small brown spots (Fig. 5). Tegmina pale brown with half of radius vein from base yellowish white and scattered with dark brown spots (Fig. 5). Coxae and anterior margin of femora dark brown (Fig. 6). Middle part of abdomen with two brown stripes (Fig. 6). Hypandrium with large brown spots (Fig. 6).

Distance between eyes about 0.4 times width of head. Ocellus slightly smaller than scrobe and ocellus width slightly narrower than interocular width (Fig. 6). Pronotum completely covering vertex, rhomboidal, smooth and impunctate (Fig. 5). Tegmina and wings well-developed, exceeding the abdomen and with posterior margin rounded (Figs 5–6). Fore femur with 7 spines along anterior margin and 2 apical spines. 1st segment of hind tarsus with plantula occupied apically and spines along most of its length.

**Male genitalia.** Supra-anal plate symmetrical, trapezoidal, posterior margin with a shallow concavity at middle, lateral sides more or less straight (Fig. 23). Paraprocts asymmetrical, left one broad, simple plate; right one with a slender finger-like protrusion whose apex bending backwards (Fig. 24). Hypandrium symmetrical, with posterior margin shallowly emarginated (Fig. 25). Apodema of complex *L1* with transverse terminal (Fig. 26). Sclerite *L2d* large and long, with a protrusion at middle (Fig. 27). Basal part of sclerite *L2vm* furcated (Fig. 27). Sclerite *R2* with apex slightly pointed (Figs 28–29).

**Male measurements.** Body length45.0 mm (including tegmen); Head length × width: 5.0 mm × 4.5 mm; Pronotum length × width: 9.0 mm × 12.5 mm; Tegmina length × width: 38.5 mm × 13.0 mm.

**Material examined.** one male, China: Guizhou Prov., Maolan Nature Preserves, 500–560m, 16–18 June 2006, coll. Yang Zaihua (SWU).

#### Distribution.

China (Guizhou); Vietnam.

#### Remarks.

This species is similar to *Pseudophoraspis fruhstorferi*, but can be distinguished by body color and male genitalia.

### Pseudophoraspis kabakovi

Anisyutkin, 1999 new record to China

http://species-id.net/wiki/Pseudophoraspis_kabakovi

Figs 7–8 30–38

Pseudophoraspis kabakovi Anisyutkin, 1999: 450.

#### Description.

Body yellowish-brown. Head yellow. Eyes dark brown, ocelli yellow (Fig. 8). Pronotum yellow with dense and small brown spots, disc brownish-yellow (Fig. 7). Tegmina yellow scattered with brown spots (Fig. 7). Anterior margin of wings with lots of brown spots. Dorsal part of abdomen brown, ventral part of abdomen yellow with dense brown spots (Fig. 8).

Vertex completely covered by pronotum (Figs 7–8). Distance between eyes about 0.15 times width of head. Ocellus same as scrobe and ocellus width equal to interocular width (Fig. 8). Pronotum rhomboidal and smooth, impunctate, with anterior margin curved and posterior margin obtusely angled (Fig. 7). Tegmina covering the abdomen totally, and apex rounded (Figs 7-8). Fore femur with 4 spines along anterior margin and one single apical spine. 1st segment of hind tarsus with spines along 2/3 of its length, plantula covering apical one third.

**Male genitalia.** Supra-anal plate rectangular, symmetrical, with posterior margin emarginated at middle (Fig. 30). Paraprocts asymmetrical, both sides with a finger-like protrusion bending backwards; the right one larger than the left (Fig. 31). Hypandrium symmetrical, with posterior margin shallowly emarginated (Fig. 32). Apodema of complex *L1* moderately sclerotized, triangular (Fig. 33). Apical outgrowth of sclerite *L2d* moderately sclerotized, short and nearly straight, basal part rough and apical part slender (Figs 34-35). Sclerite *R2* with tapering apex (Figs 36-38).

**Male measurements.** Body length 36.5 mm (including tegmen); Head length × width: 4.0 mm × 3.5 mm; Pronotum length × width: 7.5 mm × 10.5 mm; Tegmina length × width: 31.5 mm × 10.0 mm.

#### Material examined.

one male, China: Yunnan Prov., Xishuangbanna, 27-30 April 1981, coll. Zheng Zhiguang (SWU).

#### Distribution.

China (Yunnan); Vietnam.

#### Remarks.

Apodema of complex *L1* of this species is short and approximately triangular, which is obviously different from others, whose apodemas of complex *L1* are longer and approximately rectangular.

##### *Pseudophoraspis gorochovi* group

Species included here:*Pseudophoraspis recurvata* sp. n., *Pseudophoraspis incurvata* sp. n. and *Pseudophoraspis clavellata* sp. n.

### Pseudophoraspis recurvata sp. n.

urn:lsid:zoobank.org:act:0F80FAD5-60F0-490E-8C77-54048425A0A8

http://species-id.net/wiki/Pseudophoraspis_recurvata

Figs 11–12 39–48

#### Description.

Body yellowish. Head brownish yellow; vertex and interocular space testaceous; eyes dark brown, ocellus yellowish (Fig. 12). Pronotum yellow covered with dense and small brown spots, and with a few brown stripes at apex (Fig. 11). Tegmina yellowish with more or less small brown spots, and costal vein wholly white (Fig. 11). Coxae and femora ventrally scattered with small brown spots (Fig. 12). Tergite with dense and small brown dots, and a brown stripe in the middle. Sternite with slightly small brown spots (Fig. 12).

Vertex completely concealed by pronotum (Figs 11, 12). Distance between eyes about 0.2 times width of head. Ocellus same as scrobe and ocellus width equal to interocular width (Fig. 12). Pronotum broad and rhomboidal, with anterior margin curved and posterior margin obtusely produced; with punctures and two crescentic depressions on disc (Fig. 11). Tegmina and wings fully-developed, exceeding the abdomen and with apex rounded (Figs 11–12). Fore femur with 5 or 6 spines along anterior margin and one single apical spine. First segment of hind tarsus with spines along most part of its length; plantula apically occupying the terminal.

**Figures 39–65.**
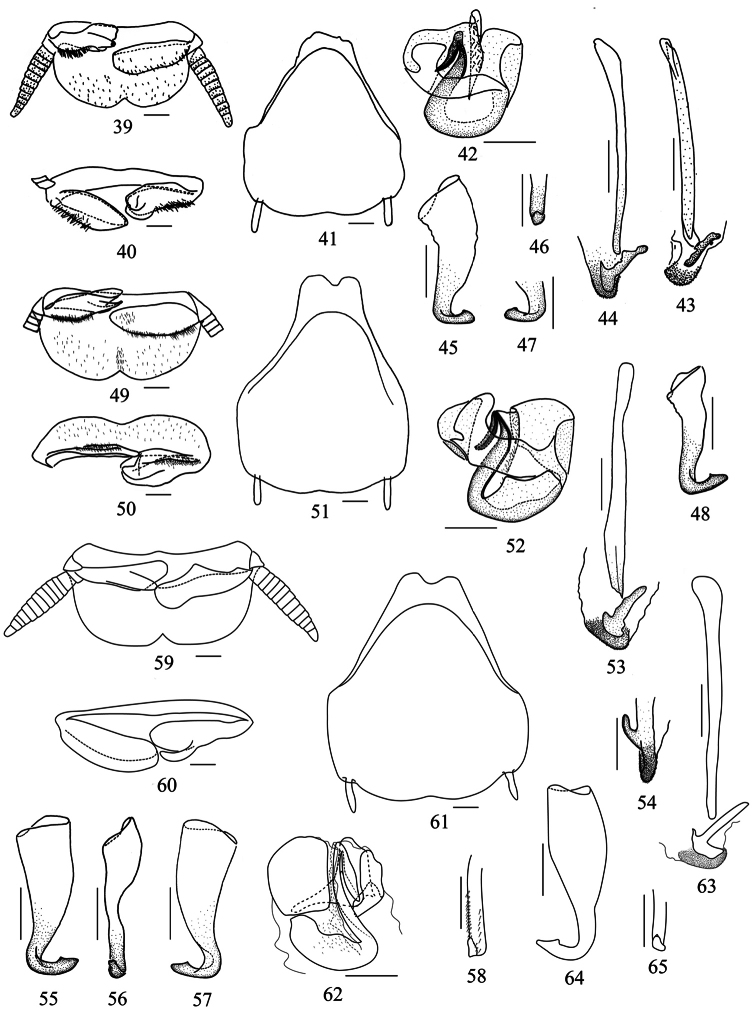
**39–48** *Pseudophoraspis recurvata* sp. n. **49–57** *Pseudophoraspis incurvata* sp. n. **58–65** *Pseudophoraspis clavellata* sp. n. **39, 49, 59** supra-anal plate, ventral view **40, 50, 60** paraproct, caudal view **41, 51, 61** hypandrium, dorsal view **42, 52, 62** complex *L1* **43, 44, 53, 63** complex *L2*, dorsal view (**43** specimen from “Hainan” **44** specimen from “Guangxi”) **54** complex *L2*, lateral view **45–48, 55–57, 64–65 ** sclerite *R2* (**45–47** specimen from “Hainan” **48** specimen from “Guangxi”) **58** 1st segment of hind tarsus. Scale bars=0.5mm.

**Male genitalia.** Supra-anal plate (Fig. 39) and hypandrium (Fig. 41) symmetrical, with posterior margin emarginate; hypandrium slightly longer than supra-anal plate. Paraprocts asymmetrical; left one broad, simple plate, right one with a finger-like protrusion bending backwards (Fig. 40). Apodema of complex *L1* short and weakly sclerotized (Fig. 42). Sclerite *L2d* moderately sclerotized, rough and weakly rugose; its apical outgrowth short, with apex slightly bending outwards (Figs 43–44). Sclerite *R2* short, with a tooth on inner margin of terminal (Figs 46-48).

**Male measurements.** Body length 26.0–27.5 mm (including tegmen); Head length × width: 3.0–3.5 mm × 2.5–3.0 mm; Pronotum length × width: 5.5–6.0 mm × 8.0–8.5 mm; Tegmina length × width: 22.0-23.0 mm × 7.5–8.0 mm.

#### Material examined.

*Holotype*, male, China: Hainan Prov., Baoting, 10 July 1959, coll. Hu Yichuan. *Paratypes*, one male, China: Hainan Prov., Baoting, 10. July 1959, coll. Hu Yichuan; one male, China: Guangxi Prov., Mt. Daqingshan, September 1958, coll. Xu Yixin.

**Distribution.** China (Hainan, Guangxi).

#### Remarks.

The species resembles *Pseudophoraspis gorochovi*, but can be distinguished by the following characters: 1) tegmina scattered with small brown spots, while the latter, tegmina lack any spots; 2) apical outgrowth of sclerite *L2d* slender, straight and with apex slightly bending outwards, the latter with apical outgrowth of sclerite *L2d* slightly bending outwards, basal part rough and terminal part slender.

#### Etymology.

The specific epithet “*recurvatus*” is derived from Latin, referring to apical outgrowth of sclerite *L2d* bending outwards.

### Pseudophoraspis incurvata sp. n.

urn:lsid:zoobank.org:act:9385822F-25A8-4519-9109-D9B0226A3B74

http://species-id.net/wiki/Pseudophoraspis_incurvata

Figs 13–14 49–57

#### Description.

Body brownish-yellow. Head yellow; occiput, vertex and interocular space brown; eyes dark brown, ocellus yellowish (Fig. 14). Pronotum yellow scattered with small brown spots and some brown stripes near posterior margin (Fig. 13). Tegmina yellow, costal vein wholly yellowish, basal part scattered with small brown spots and the rest with large brown spots (Fig. 13). Anterior margin of wings covered with a few brown spots. Anterior margin of femur brown; coxa and femur scattered with small brown spots (Fig. 14). Abdomen densely scattered with brown spots, of which on tergites are denser than that of on sternites (Fig. 14). Tergites with a brown stripe in the middle (Fig. 13).

Vertex completely concealed by pronotum (Figs 13–14). Distance between eyes about 0.2 times width of head. Ocellus same as scrobe and ocellus width equal to interocular width (Fig. 14). Pronotum punctured and with two crescentic depressions on disc, rhomboidal, broader than long; with anterior margin curved and posterior margin obtusely produced (Fig. 13). Tegmina and wings fully-developed, apex rounded (Figs 13–14). Fore femur with 5 or 6 spines along anterior margin and 2 apical spines. First segment of hind tarsus with spines along most of its length and plantula occupying the terminal. Each tergite with a small ridge in the middle.

#### Male genitalia.

Supra-anal plate symmetrical, semicircular, emarginated along posterior margin (Fig. 49). Paraproct asymmetrical; left one broad, simple plate, right one with a finger-like protrusion bending backwards (Fig. 50). Hypandrium symmetrical, shallowly emarginate in middle of posterior margin (Fig. 51). Hypandrium slightly longer than supra-anal plate. Apodema of complex *L1* short (Fig. 52). Sclerite *L2d* small, moderately sclerotized, apical outgrowth of sclerite *L2d* bending inwards (Figs 53–54). Sclerite *R2* with apex pointed (Figs 55–57).

**Male measurements.** Body length 29.5 mm (including tegmen); Head length × width: 3.6 mm × 3.0 mm; Pronotum length × width: 6.5 mm × 9.5 mm; Tegmina length × width: 24.5 mm × 8.5 mm.

#### Material examined.

*Holotype*, male, China: Hainan Prov., Mt. Jianfengling, 31 July 1982, coll. Chen Zhiqing (SWU).

#### Distribution.

China (Hainan).

#### Remarks.

The species is similar to *Pseudophoraspis recurvata* sp. n., but can be distinguished by characters as follows: 1) the body larger than that of the latter, 2) apical outgrowth of sclerite *L2d* rough and bending inwards, while the latter straight and with apex slightly bending outwards.

#### Etymology.

The specific name is from the Latin word “*incurvatus*”, referring to apical outgrowth of sclerite *L2d* bending inwards.

### Pseudophoraspis clavellata sp. n.

urn:lsid:zoobank.org:act:D441247B-3568-4AE5-ACDF-562E75F8A8FC

http://species-id.net/wiki/Pseudophoraspis_clavellata

Figs 9–10 58–65

#### Description.

Body brownish-yellow. Head yellow; vertex and interocular space brown; eyes dark brown, ocelli yellowish (Fig. 10). Pronotum yellow scattered with small brown spots and some brown stripes near posterior margin (Fig. 9). Tegmina variegated scattered with brown spots, costal vein almost completely yellowish (Fig. 9). Anterior margin of wings scattered with few brown spots. Anterior margin of femur brown. Coxa, femur, and abdomen scattered with small brown spots (Fig. 10).

Vertex completely covered by pronotum. Distance between eyes at vertex is 0.3 times the width of head. Ocellus oval, much smaller than scrobes and ocellus width equal to interocular width (Fig. 10). Pronotum rhomboidal and with anterior margin curved, posterior margin obtusely produced; with punctures and two crescentic depressions on disc (Fig. 9). Tegmina and wings fully-developed, apex rounded (Figs 9–10). Fore femur with 4 or 6 spines along anterior margin and 1 apical spine. First segment of hind tarsus with 2 rows of spines along most of its length and plantula occupying the terminal (Fig. 58).

#### Male genitalia.

Supra-anal plate (Fig. 59) and hypandrium (Fig. 61) symmetrical, distinctly shorter than hypandrium; both emarginate along posterior margin. Paraprocts asymmetrical; left one broad, simple plate, right one with a finger-like protrusion bending backwards (Fig. 60). The apodema of complex *L1* short, median emargination long (Fig. 62). Apical outgrowth of sclerite *L2d* more or less straight and slender (Fig. 63). Sclerite *R2* with apex pointed and a tooth on inner margin (Figs 64–65).

**Male measurements.** Body length 31.5 mm (including tegmen); Head length × width: 3.5 mm × 2.6 mm; Pronotum length × width: 7.0 mm × 9.5 mm; Tegmina length × width: 26.5 mm × 8.5 mm.

#### Material examined.

*Holotype*, male, China: Yunnan Prov., Xishuangbanna, 11–13 May 1974, coll. Chou Io & Yuan Feng (NWAFU).

#### Distribution.

China (Yunnan).

#### Remarks.

*Pseudophoraspis clavellata* sp. n. is similar to *Pseudophoraspis incurvata* sp. n., but can be distinguished by the following characters: 1) ocellus small (larger in *Pseudophoraspis incurvata* sp. n.); 2) tegmina variegated with irregular brown spots (tegmina yellowish brown scattered with regular and round brown spots in *Pseudophoraspis incurvata* sp. n.); 3) apical outgrowth of sclerite *L2d* more or less straight and slender (bending inwards and with apex pointed in *Pseudophoraspis incurvata* sp. n.).

#### Etymology.

The specific epithet is derived from the Latin word “*clavellatus*”, referring to apical outgrowth of sclerite *L2d* being nearly straight and slender.

## Supplementary Material

XML Treatment for Pseudophoraspis

XML Treatment for Pseudophoraspis fruhstorferi

XML Treatment for Pseudophoraspis tramlapensis

XML Treatment for Pseudophoraspis kabakovi

XML Treatment for Pseudophoraspis recurvata

XML Treatment for Pseudophoraspis incurvata

XML Treatment for Pseudophoraspis clavellata
